# Parametric Images in Assessing Bone Grafts Using Dynamic ^18^F-Fluoride PET

**DOI:** 10.1155/2011/189830

**Published:** 2011-05-22

**Authors:** Lingfeng Wen, Stefan Eberl, (David) Dagan Feng, Paul Stalley, Gang Huang, Michael J. Fulham

**Affiliations:** ^1^Department of PET and Nuclear Medicine, Royal Prince Alfred Hospital, Sydney, NSW 2050, Australia; ^2^School of Information Technologies, University of Sydney, Sydney, NSW 2006, Australia; ^3^Department of Electronic and Information Engineering, The Hong Kong Polytechnic University, Kowloon, Hong Kong; ^4^Med-X Research Institute, Shanghai JiaoTong University, Shanghai 200030, China; ^5^Department of Orthopedic Surgery, Royal Prince Alfred Hospital, Sydney, NSW 2050, Australia; ^6^Faculty of Medicine, University of Sydney, Sydney, NSW 2006, Australia

## Abstract

The early identification of graft failure would improve patient management. ^18^F-fluoride is a suitable tracer for quantifying bone metabolism. Performance of parametric images constructed by Patlak graphical analysis (PGA) with various time periods was evaluated in the analysis of dynamic ^18^F-fluoride PET studies of eight patients with fibula bone grafts after limb salvage surgery. The PGA parametric image approach tended to underestimate influx rate. The linear portion of PGA analysis was found to be from 10 to 50 min. It shows promise in providing a quantitative assessment of the viability of bone grafts.

## 1. Introduction

Free vascularised fibula grafting is well described in limb salvage surgery after resection of bone tumours. Nonunion at the osteotomy site and fracture of the graft are recognised complications, which may be related to poor blood flow within the graft. Currently there is no diagnostic modality that can reliably assess graft viability. Graft viability is usually judged by evidence of bony bridging and graft hypertrophy on plain radiographs, but this is problematic and delayed union and infection cannot be reliably predicted. The early identification of graft failure would improve patient management, and a reliable assessment of graft viability would also help identify characteristics associated with successful grafts. 

Functional imaging techniques, such as positron emission tomography (PET), can visualise subtle metabolic changes and have the potential for assessing graft viability. The PET tracer ^18^F-fluoride has been used to evaluate regional bone metabolism and skeletal kinetics [1–5] as well as monitoring therapeutic response [6]. The dynamic behaviour of complex biological systems can be described by suitable kinetic models in functional imaging. Rate constants and macroparameters of kinetic models are related to biological processes; hence, these parameters could be used to aid clinical evaluation. 

Kinetic modelling of PET imaging usually involves obtaining a series of blood samples for the input function (IF), and a tissue time activity curve (TTAC), derived from dynamic imaging data as the output function. Rate constants are determined by parameter estimation methods, which fit the TTAC according to an underlying kinetic model. Traditionally, manually defined regions of interest (ROI) are used to derive average, but reduced noise, TTACs for the selected regions by deriving the mean activities for the defined ROI. Alternatively, parameter estimation can be conducted voxel by voxel to form a three-dimensional parametric image volume whose voxel values represent quantitative functional parameters. The parametric image approach reduces operator-dependant bias in the manual delineation of ROI and eliminates the need to know the spatial distribution of newly developed tracers [7]. Furthermore, the parametric image approach readily visualizes the spatial distribution of quantitative functional parameters. 

The nonlinear least square (NLS) analysis, a standard parameter estimation method, provides estimates with optimum statistical accuracy in kinetic analysis by iteratively adjusting the kinetic parameters of the nonlinear model equations [8]. There is intrinsic noise in functional imaging data, thus an appropriate weight function is usually chosen to derive the objective function in NLS fitting. Thus, NLS is often referred to as the weighted nonlinear least square (WNLS). However, NLS/WNLS are not suitable for the construction of parametric images due to their high computational cost, proneness to be trapped in local minima, and a requirement for appropriate initial parameters. Graphical analysis (GA) methods, such as the Patlak (PGA) [9] and Logan approaches [10], transform kinetic model equations into a simple linear plot of selected variables. The slope and intercept of GA methods are usually related to parameters of interest. Computational efficiency and reliable parameter estimates make GA methods suitable for deriving parametric images. 

A number of investigators reported on the kinetic analysis of ^18^F-fluoride in the evaluation of vascularisation of allogenic bone grafts [11–16]. Different kinetic quantitative approaches are also reported [5, 12, 15]. However, these comparisons were only limited to the TTACs derived by manually placing ROI on the images, which is time consuming and prone to subjective bias. Thus, the performance of these techniques for generating three-dimensional parametric images has not been fully evaluated. In addition, there is no consensus on the optimum time period in defining the linear portion of the PGA plot, which may also introduce bias in PGA plots. 

Our aim was to systematically investigate the performance of parametric images derived by the PGA method and evaluate the optimum linear portion of PGA using dynamic ^18^F-fluoride PET imaging. Eight patients with limb salvage surgery and bone grafting were included with volume of interest (VOI) defined on bone grafts and normal bone tissues. Quantitative rate constants and net influx rates were also derived for VOI-derived TTACs with NLS and PGA analysis for comparison. 

## 2. Materials and Methods

### 2.1. Kinetic Model for Fluoride Bone Metabolism

**Figure 1:**
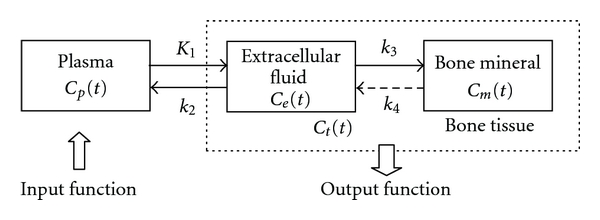
Three-compartment and four-parameter kinetic model for fluoride bone metabolism.

A three-compartment and four-parameter model has been used in the kinetic analysis for fluoride bone metabolism (Figure 1) [1]. It consists of plasma, extracellular fluid, and bone mineral compartments. Rate constants describe the transport of ^18^F-fluoride between the compartments with *K*_1_ representing the unidirectional clearance of fluoride from plasma to bone tissue, *k*_2_ the reverse transport of fluoride from bone tissue to plasma, *k*_3_ reflecting the incorporation, and *k*_4_ the release of fluoride from the bone mineral compartment. The unit of *K*_1_ is mL·min^−1^ *·*mL^−1^, while *k*_2_, *k*_3_, and *k*_4_ have units of min^−1^.

*C*_*p*_(*t*) represents the plasma concentration of ^18^F-fluoride, while *C*_*e*_(*t*) and *C*_*m*_(*t*) are ^18^F-fluoride concentrations in extracellular fluid and bone. *C*_*t*_(*t*) is the sum of *C*_*e*_(*t*) and *C*_*m*_(*t*) and represents total concentration of ^18^F-fluoride in bone tissue. Through kinetic modelling technique, the output function, *C*_*t*_(*t*), can be represented by the input function, *C*_*p*_(*t*), in the following:

(1) $$\begin{array}{cc} C_{t}\left(t\right) & =\frac{K_{1}}{\alpha_{2}-\alpha_{1}}\left[\left(k_{4}-\alpha_{1}\right)\cdot e^{-\alpha_{1}t}+\left(\alpha_{2}-k_{4}\right)\cdot e^{-\alpha_{2}t}\right]\\ & \;⊗C_{p}\left(t\right)+\frac{K_{1}k_{3}}{\alpha_{2}-\alpha_{1}}\left(e^{-\alpha_{1}t}-e^{-\alpha_{2}t}\right)⊗C_{p}\left(t\right), \end{array}$$

where $\alpha_{1,2}=\left(k_{2}+k_{3}+k_{4}\mp \sqrt{{\left(k_{2}+k_{3}+k_{4}\right)}^{2}-4k_{2}k_{4}}\right)/2\;\text{and}\;⊗$ denotes the mathematical convolution operator.

For the ROI-based approach, a fifth parameter of fractional blood volume (fBV) is often included in parameter estimation to address spillover from capillary blood activity within the tissue regions as shown in

(2) $$\begin{array}{cc} C_{t}\left(t\right) & =\left(1-\text{fBV}\right)\cdot \frac{K_{1}}{\alpha_{2}-\alpha_{1}}\\ & \;\times \left[\left(k_{4}-\alpha_{1}\right)\cdot e^{-\alpha_{1}t}+\left(\alpha_{2}-k_{4}\right)\cdot e^{-\alpha_{2}t}\right]\\ & \;⊗C_{p}\left(t\right)+\frac{K_{1}k_{3}}{\alpha_{2}-\alpha_{1}}\left(e^{-\alpha_{1}t}-e^{-\alpha_{2}t}\right)\\ & \;⊗C_{p}\left(t\right)+\text{fBV}\cdot C_{p}\left(t\right). \end{array}$$

The influx rate macroparameter, *K*_*i*_, represents net influx or uptake of plasma fluoride activity by the bone matrix and has units of mL*·*min^−1^ *·*mL^−1^ and is given by

(3) $$\begin{array}{c} K_{i}=K_{1}\times \frac{k_{3}}{k_{2}+k_{3}}. \end{array}$$

*K*_*i*_ is an important macroparameter for describing the level of osteoblastic activity and is a measure of bone metabolism.

### 2.2. Image Acquisition

The studies and protocols were approved by the Ethics Committee of the Central Sydney Area Health Service. Eight patients with previous limb salvage surgery and bone grafting were studied; there were 3 men and 5 women with age range of 20–53 years. The bone grafts were taken from the fibula. The time between the bone graft surgery and PET scans was ranging from 1.3 to 4.9 years. All imaging studies were carried out on an ECAT 951R whole body PET scanner (Siemens/CTI, Knoxville, Tenn, USA) in Royal Prince Alfred Hospital. The scanner acquired 31 planes simultaneously with a slice separation of 3.375 mm and axial field of view (FOV) of 10.8 cm. 206.2 ± 18.1 MBq ^18^F-sodium fluoride was infused at a constant rate over a 3-minute period. Simultaneously with the beginning of tracer infusion, a 60-minute dynamic sequence of 29 emission scans was started with twelve 10-second, six 30-second, and eleven 5-minute frames. A postinjection transmission method was used to correct for photon attenuation [17].

12 arterialised-venous blood samples [8, 18] were obtained every 30 s for the first 6 min after injection, followed by 4 samples at 1-minute, 2 samples at 5-minute, and 4 samples at 10-minute intervals. Plasma samples were counted in a *γ*-well-counter cross-calibrated with the PET scanner.

Transmission images were reconstructed by an ordered-subset median-root-prior (OS-MRP) iterative reconstruction algorithm with 2 iterations and 16 subsets, which were then segmented into lung, bone, and soft tissue. The dynamic emission images were reconstructed into 128 × 128 matrices by an ordered-subset expectation-maximization (OS-EM) iterative reconstruction technique with 1 iteration and 16 subsets. Attenuation correction was implemented in the OS-EM reconstruction with the attenuation coefficients derived from the segmented reconstructed transmission images.

### 2.3. VOI Delineation

**Figure 2:**
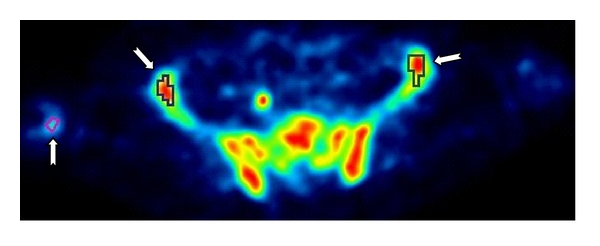
Example of one transaxial plane from the average images of the last six frames. The arrows indicate manually drawn ROI.

The images from the last six frames, ranging from 30 to 60 min, were averaged to aid definition of VOI, which were defined using the PMOD package (version 3.1, PMOD Technologies Ltd., Zurich, Switzerland) by manually delineating ROIs on voxels with similar values across a sequence of image planes shown in Figure 2. VOIs were defined on the bone graft and also on normal bone in the ilium and lumbar vertebra. The intervertebral discs were not included in the VOIs for lumbar vertebra. 

**Figure 3:**
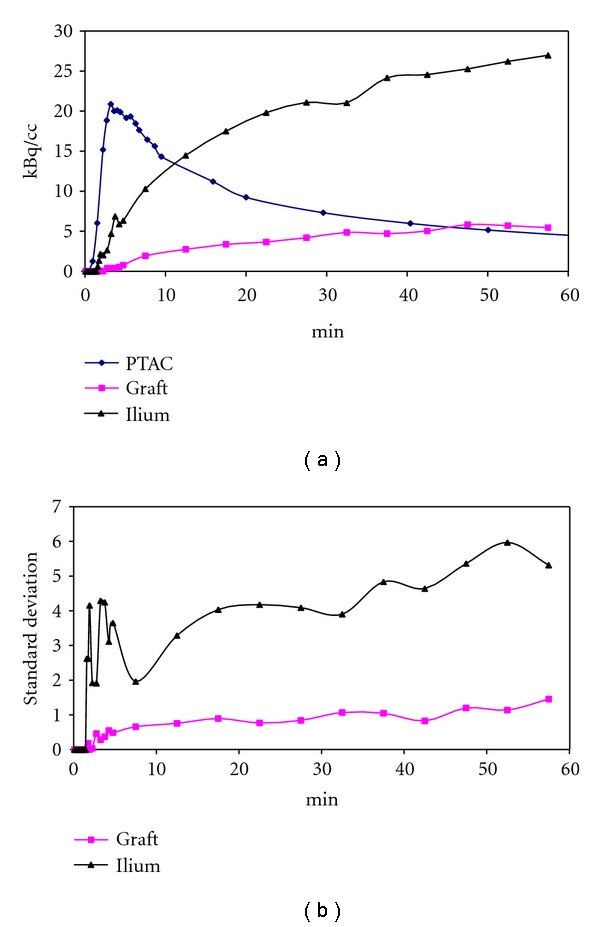
(a) PTAC and TTACs for graft and ilium. (b) Standard deviation curves.

Mean value and standard deviation (SD) value were then derived for each VOI on each frame of reconstructed images to form TTAC and SD curve.  Figure 3 plots typical TTACs and plasma time activity curve (PTAC) of one patient in Figure 3(a) and SD curves over time in Figure 3(b). 

### 2.4. Constructing Parametric Images

The Patlak graphical analysis (PGA) assumes a three-compartment and three-parameter model, where the release of fluoride from the bony matrix is considered negligible, that is, *k*_4_ = 0 [9]. This leads to (4) to describe the relations between the input and output function in Figure 1:

(4) $$\begin{array}{c} \frac{C_{t}\left(t\right)}{C_{p}\left(t\right)}=\frac{K_{1}\cdot k_{3}}{k_{2}+k_{3}}\cdot \frac{\int_{0}^{t}C_{p}\left(\tau \right)d\tau }{C_{p}\left(t\right)}+\frac{k_{2}}{k_{2}+k_{3}}\frac{C_{e}\left(t\right)}{C_{p}\left(t\right)}. \end{array}$$

When equilibrium has been reached after a sufficient time (*t* > t*), (4) can be simplified to (5), where *Const* represents a constant value:

(5) $$\begin{array}{c} \frac{C_{t}\left(t\right)}{C_{p}\left(t\right)}\approx K_{i}\cdot \frac{\int_{0}^{t}C_{p}\left(\tau \right)d\tau }{C_{p}\left(t\right)}+Const,\;t>t^{*}, \end{array}$$

*K*_*i*_ can then be readily derived from the slope of the linear plot of ∫_0_^*t*^ *C*_*p*_(*τ*)*dτ*/*C*_*p*_(*t*) versus *C*_*t*_(*t*)/*C*_*p*_(*t*). 

PGA was used to derive the parametric images of *K*_*i*_ according to (5) by an in-house package implemented in the IDL language (version 6.0, ITT Visual Information Solutions, Boulder, Colo, USA). Four linear periods of the PGA plot reported in the literature were used to derive *K*_*i*_ voxel by voxel for 4 to 60 min [6], 10 to 50 min [12, 15], 20 to 50 min [11], and 20 to 60 min [13, 14]. The value of a voxel was set to zero if the derived *K*_*i*_ was negative.

For comparison, the derived VOIs in Section 2.3 were used to derive mean and SD values of *K*_*i*_ from the parametric images, respectively, for the same patient. PGA was also applied to derive *K*_*i*_ for VOI-derived TTACs with the same linear periods investigated as in the parametric images.

### 2.5. NLS Analysis

The weights used in NLS analysis were defined by the inverse of the VOI-derived SD curve. The range of parameter variation was set from 0 to 1 for fBV, *K*_1_, *k*_2_, and *k*3 and 0 to 0.1 for *k*_4_, while the initial parameters were 0.1 mL*·*mL^−1^, 0.1 mL*·*min^−1^ *·*mL^−1^, 0.15 min^−1^, 0.1 min^−1^, and 0.01 min^−1^ for fBV, *K*_1_, *k*_2_, *k*_3_, and *k*_4_ which were specified [12]. The generated TTACs were fitted by using the Marquardt-Levenberg algorithm to adjust the rate constant according to (2) using the PMOD package. The maximum number of iterations was set at 200.

For the equivalent comparison with the PGA method, NLS analysis was also applied to fit the VOI-derived TTAC with fBV and *k*_4_ predefined to be 0.

## 3. Results

### 3.1. Effect of Parametric Images

**Figure 4:**
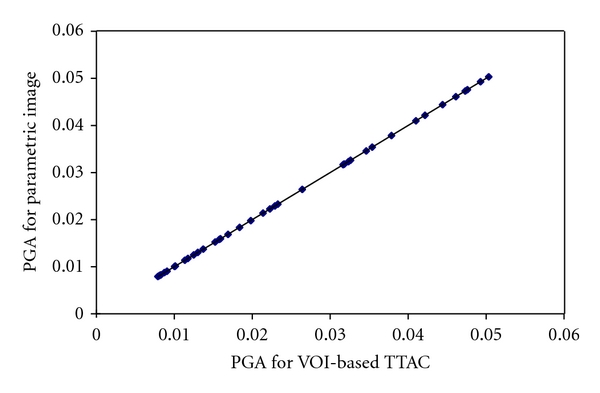
Plots of values of *K*_*i*_ derived by PGA from VOI-based TTAC fitting and values derived from VOI placed on the parametric images. The linear fitting period was 10 to 50 min.

Figure 4 plots the net influx rates derived from VOI-based TTACs versus those from the parametric images by PGA with 10 to 50 min selected as the linear fitting period. A significant, high correlation (*r* > 0.999) was observed between the values of parametric images and values of VOI-based TTAC for the four PGA approaches. 

Comparably, low values of SD were observed in all the regions for parametric image of *K*_*i*_ (Table 1). This indicated that PGA provided reliable and consistent estimates of *K*_*i*_ for region averaged TTACs as well as voxel-by-voxel TTACs.

**Figure 5:**
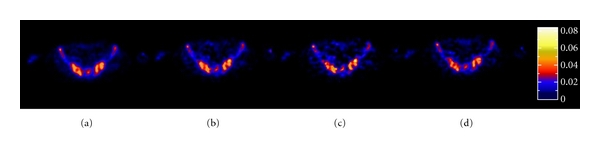
Parametric images of *K*_*i*_ derived by PGA with the linear portion of (a) 4–60 min, (b) 10–50 min, (c) 20–50 min, and (d) 20–60 min, for the transaxial plane of the average image shown in Figure 2.

Figure 5 shows the parametric images derived by PGA approach for the investigated linear fitting periods. More detail is achieved by the PGA with the linear period of 20–50 min (Figure 5(c)) and 20–60 min (Figure 5(d)). However, this comes at the expense of increased noise and slightly less reliable parameter estimation reliability. 

### 3.2. Rate Constants of NLS Analysis

Table 2 lists the rate constants derived by NLS for the three-compartment and five-parameter model as well as the relevant influx rate (*K*_*i*−5*p*_). The mean values of parametric images of *K*_*i*_ derived by PGA are also listed for the comparison. The influx rate for three-compartment and three-parameter model with fBV = 0 and *k*_4_ = 0 is also given in the table and is referred to as *K*_*i*−3*p*_. 

Some unsuccessful fits were observed by NLS analysis such as when *k*_2_ was close to zero or one. This is not unexpected because the success of physiological meaningful NLS analysis is dependent on not only appropriate initial parameters but also proper noise model and underlying kinetic model. Insufficient data quality in TTAC may give rise to such unsuccessful fit for the three-compartment and five-parameter kinetic model. Interestingly, most of estimated fBV is observed to be zero, which implies that the spillover contribution from surrounding vascular system can be ignored.

Table 3 lists the mean values and SD for all the VOIs with unsuccessful fits excluded. The lowest value of *K*_1_ was observed for the bone grafts, which implies reduced blood flow. Similar values of *k*_2_, *k*_3_, and *k*_4_ were observed in the graft, ilium, and lumbar vertebra. The lower *K*_1_ of bone graft in contrast to normal bone suggests that reduced osteoblastic activity, indicated by reduced *K*_*i*_, of the bone graft was mainly caused by poor vascularisation and reduced blood flow. This is consistent with the findings in [13]. 

### 3.3. Comparison of NLS and PGA for Parametric Image

**Figure 6:**
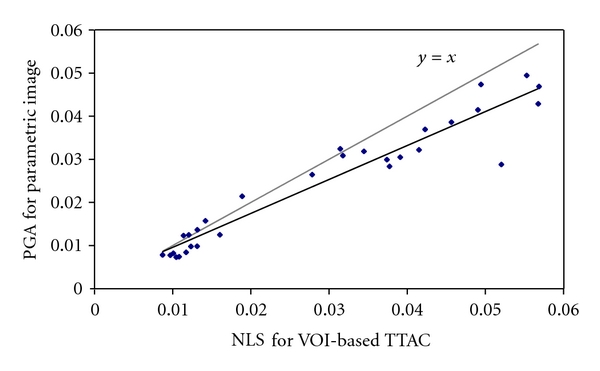
Linear regression analysis between NLS for VOI-based TTAC and PGA for parametric image with linear period of 20 to 60 min. Black line: the plot of obtained linear regression; grey line: theoretical ideal linear regression.

Linear regression analysis was applied to compare the accuracy of the net influx rate derived by PGA compared with NLS for the three-compartment and five-parameter model with unsuccessful fits excluded.  Table 4 lists the results of linear regression for the studied four PGA linear periods. One example of linear regression analysis is plotted in Figure 6 for the PGA with the linear period of 20 to 60 min.

A high linear correlation was observed for *K*_*i*_ derived from PGA parametric images compared with NLS with the linear correlation *r* ≥ 0.961. However, PGA tends to underestimate *K*_*i*_ with the slope of linear analysis ranging from 0.787 to 0.890 as compared with *K*_*i*−5*p*_ derived by NLS. The values of *K*_*i*−3*p*_ in Table 2 are almost identical to the values of original *K*_*i*−5*p*_ when fitted *k*_4_ is 0, while *K*_*i*−3*p*_ is lower than *K*_*i*−5*p*_ when fitted *k*_4_ is >0. The same underlying model is assumed in deriving *K*_*i*−3*p*_ for NLS and *K*_*i*_ for PGA. Ignoring the contribution of *k*_4_ is likely to be the main reason for the underestimation of the influx rate with PGA.

The potential for underestimating *K*_*i*_ with PGA should be recognised when interpreting the kinetic analysis of bone metabolism. However, the simplicity of PGA and the benefit of parametric images make PGA a more attractive approach for the quantitative analysis of bone metabolism. Overall, taking reliability, accuracy, and linear regression analysis into account, the PGA analysis with the linear period of 10 to 50 min should provide best parametric images of *K*_*i*_ in the quantitative analysis of bone metabolism.

## 4. Discussion

^18^F-fluoride PET with quantitative kinetic analysis has allowed the quantitative assessment of regional lesions and treatment response in metabolic bone disease and also enabled early identification of bone viability and discriminated uneventful and impaired healing processes of fractures, bone grafts, and osteonecrosis [19]. Despite its desirable characteristics of high and rapid bone uptake with very rapid blood clearance, ^18^F-fluoride PET scanning has yet to become part of routine clinical practice [20].

The Gaussian noise model is usually assumed in the NLS fitting with the weights defined according to the activity of TTAC and the duration of imaging frame. In this study, the weight is defined by the inverse of the VOI-derived standard deviation instead, which provides an approximate estimation of noise distribution for the VOI-derived TTAC. However, due to the low signal-to-noise ratio in early, short imaging frames, the iterative reconstruction caused the values of voxels with low activity to be estimated to be zero and explains why the values of SD are shown to be zero for the early frames in Figure 3(b). Thus, the extraordinary high weights assigned for the early zero points would give rise to unsuccessful NLS fits, especially for the lower ^18^F-fluoride uptake in the grafts. With the first sixteen data points (first 4 min) excluded, NLS analysis was found to achieve successful fitting for VOI-based TTACs as summarised in Table 2. For example, the rate constants (region no. 7, graft region in Figure 3) are 0.000 for fBV, 0.025 for *K*_1_, 0.179, 0.173, and 0.006, respectively, for *k*_2_, *k*_3_, and *k*_4_ while *K*_*i*−5*p*_ is 0.012, which is identical to the mean value of influx rate derived from the corresponding parametric images VOI. 

The estimation of *K*_1_ in NLS analysis was sensitive to data points in early imaging time. The obtained zero points in the first 4-minute period may lead to biased estimation of *K*_1_. However, the parameter of interest, *K*_*i*_, was insensitive to the early imaging time. The NLS analysis was expected to provide reasonable estimate of *K*_*i*_ as the reference in the evaluation of PGA method.

The late time period inherently used for the PGA fit avoids the problem of the low count early frames for the PGA method. We observed high reliability for the studied PGA with four linear periods in the generated parametric images of *K*_*i*_, which are highly correlated with NLS-derived *K*_*i*_ values. Interestingly, the underestimation of PGA-derived *K*_*i*_ tends to be higher with later starting times of the linear period. Thus, the inclusion of early staring time in PGA analysis is necessary to reduce the underestimation of *K*_*i*_ due to the simplifying assumptions of the underlying kinetic model.

Hybrid PET/CT scanner has now become widespread with improved image quality and shorter imaging time with coregistered CT images. Combining with the detailed anatomical information readily available from CT images, the state-of-the-art PET/CT imaging may lead to more accurate assessment of graft viability. This requires in-depth investigation on improving the accuracy of CT-based attenuation correction for PET scans, especially for bone and graft tissues, to take advantage of the benefits offered by the hybrid scanners.

## 5. Conclusions

Dynamic ^18^F-fluoride PET imaging studies in patients with limb salvage surgery and fibula bone grafts were analysed using NLS and PGA methods to derive TTACs and parametric images. 39 regions were analysed respectively for the bone graft, ilium, and lumbar vertebrae. The results show that parametric images derived by PGA are consistent with VOI-based parameter estimation by PGA with high reliability. The PGA approach tended to underestimate influx rate because the assumption that *k*_4_ = 0 was not valid in all cases. The linear portion for PGA analysis was suggested to be from 10 to 50 min. This quantitative approach using dynamic ^18^F-fluoride PET imaging allows the assessment of bone metabolism, and additional clinical studies will clarify its role in identifying bone graft viability.

## Figures and Tables

**Table 1:** Summary of standard deviation (mean ± SD) in all the regions for parametric images of *K*_*i*_.

Linear Period	4–60 min	10–50 min	20–50 min	20–60 min
Mean ± SD	0.004 ± 0.002	0.005 ± 0.003	0.007 ± 0.004	0.005 ± 0.003

**Table 2:** Kinetic parameters for bone metabolism.

	No.	fBV	*K*_1_	*k*_2_	*k*_3_	*k*_4_	*K*_*i*−5*p*_	*K*_*i*−3*p*_	*K*_*i*_^4–60^	*K*_*i*_^10–15^	*K*_*i*_^20–50^	*K*_*i*_^20–60^
Graft	1	0.000	0.037	0.118	0.091	0.016	0.016	0.002^#^	0.014	0.016	0.016	0.012
	2	0.000	0.051	0.389	0.120	0.000	0.012	0.012	0.013	0.013	0.013	0.012
	3	0.000	0.018	0.060	0.098	0.000	0.011	0.012	0.012	0.011	0.011	0.012
	4	0.084	0.013	0.000^#^	0.453	0.006	0.013	0.012	0.012	0.012	0.012	0.011
	5	0.042	0.016	0.000^#^	1.000^#^	0.100	0.016	0.016	0.016	0.016	0.017	0.018
	6	0.000	0.044	0.064	0.048	0.000	0.019	0.020	0.022	0.021	0.022	0.021
	7	0.000	0.012	0.000^#^	0.000^#^	0.000^#^	0.000^#^	0.011	0.011	0.012	0.012	0.010
	8	0.000	0.057	0.030	0.001	0.000	0.002^#^	0.027	0.029	0.026	0.026	0.027
	9	0.000	0.007	0.000^#^	0.910	0.090	0.007^#^	0.007^#^	0.022	0.023	0.022	0.015
	10	0.000	0.014	0.000^#^	0.000^#^	0.000	0.006^#^	0.013^#^	0.023	0.022	0.020	0.018
	11	0.000	0.070	0.844	0.136	0.006	0.010	0.008	0.008	0.009	0.010	0.008
	12	0.009	0.120	0.444	0.044	0.008	0.011	0.009	0.008	0.008	0.009	0.007
	13	0.000	0.032	0.054	0.037	0.004	0.013	0.012	0.015	0.015	0.015	0.014

Ilium	14	0.000	0.172	0.486	0.135	0.007	0.037	0.033	0.033	0.032	0.030	0.030
	15	0.000	0.125	0.229	0.078	0.004	0.032	0.030	0.033	0.033	0.034	0.031
	16	0.000	0.248	0.302	0.062	0.006	0.042	0.038	0.045	0.044	0.041	0.037
	17	0.000	0.408	0.895	0.122	0.004	0.049	0.044	0.045	0.048	0.047	0.041
	18	0.000	0.180	0.220	0.074	0.007	0.046	0.040	0.044	0.042	0.040	0.039
	19	0.000	0.234	0.592	0.170	0.014	0.052	0.039	0.040	0.041	0.034	0.029
	20	0.048	0.067	0.079	0.126	0.015	0.042	0.035	0.035	0.035	0.034	0.032
	21	0.000	0.145	0.212	0.131	0.006	0.055	0.050	0.052	0.049	0.048	0.049
	22	0.002	0.136	0.484	0.165	0.003	0.034	0.032	0.032	0.032	0.032	0.032
	23	0.063	0.110	0.069	0.074	0.008	0.057	0.052	0.052	0.050	0.048	0.047
	24	0.000	0.012	0.000^#^	0.999	0.000	0.012^#^	0.011^#^	0.020	0.018	0.018	0.020
	25	0.024	0.067	1.000^#^	0.609	0.009	0.025	0.022	0.020	0.020	0.018	0.018
	26	0.000	0.075	0.197	0.216	0.013	0.039	0.029	0.033	0.035	0.035	0.030
	27	0.000	0.024	0.016	0.023	0.000	0.014	0.014	0.017	0.017	0.017	0.016
	28	0.000	0.036	0.312	0.098	0.004	0.009	0.008	0.008	0.008	0.008	0.008
	29	0.000	0.039	0.337	0.170	0.013	0.013	0.010	0.011	0.010	0.009	0.010
	30	0.000	0.058	0.801	0.167	0.008	0.010	0.008	0.009	0.008	0.008	0.008
	31	0.000	0.035	0.326	0.178	0.011	0.012	0.010	0.011	0.010	0.010	0.010
	32	0.000	0.108	0.171	0.070	0.000	0.031	0.032	0.034	0.032	0.032	0.032
	33	0.000	0.024	0.000^#^	0.090	0.090	0.024^#^	0.014^#^	0.035	0.038	0.038	0.032

Lumbar vertebra	34	0.065	0.090	0.106	0.048	0.000	0.028	0.027	0.029	0.023	0.021	0.026
	35	0.003	0.163	0.226	0.121	0.011	0.057	0.046	0.048	0.046	0.043	0.043
	36	0.000	0.146	0.175	0.090	0.005	0.049	0.042	0.050	0.047	0.045	0.047
	37	0.000	0.103	0.283	0.163	0.010	0.038	0.031	0.031	0.032	0.033	0.028
	38	0.000	0.037	0.513	0.201	0.014	0.010	0.008	0.008	0.008	0.007	0.007
	39	0.000	0.040	0.491	0.201	0.013	0.012	0.009	0.009	0.009	0.008	0.008

^#^Unsuccessful fit.

**Table 3:** Mean and standard deviation (mean ± SD) of derived rate constants for successful NLS analysis.

	fBV	*K*_1_	*k*_2_	*k*_3_	*k*_4_	Flux-*K*_*i*_
Graft	0.001 ± 0.003	0.053 ± 0.034	0.282 ± 0.297	0.082 ± 0.039	0.005 ± 0.006	0.013 ± 0.003
Ilium	0.007 ± 0.003	0.129 ± 0.076	0.337 ± 0.276	0.121 ± 0.042	0.007 ± 0.003	0.034 ± 0.014
Lumbar vertebra	0.011 ± 0.026	0.097 ± 0.052	0.299 ± 0.168	0.137 ± 0.062	0.009 ± 0.005	0.032 ± 0.019

**Table 4:** Linear regression between NLS and PGA for parametric images.

Linear portion	4–60 min	10–50 min	20–50 min	20–60 min
*r*	0.979	0.983	0.970	0.961
*y* = *ax* + *b *	0.890 × *K*_*i*−5*p*_ + 0.001	0.876 × *K*_*i*−5*p*_ + 0.001	0.818 × *K*_*i*−5*p*_ + 0.002	0.787 × *K*_*i*−5*p*_ + 0.002
